# Examining the Clinical Prognosis of Critically Ill Patients with COVID-19 Admitted to Intensive Care Units: A Nationwide Saudi Study

**DOI:** 10.3390/medicina57090878

**Published:** 2021-08-26

**Authors:** Abbas Al Mutair, Alyaa Elhazmi, Saad Alhumaid, Gasmelseed Y. Ahmad, Ali A. Rabaan, Mohammed A. Alghdeer, Hiba Chagla, Raghavendra Tirupathi, Amit Sharma, Kuldeep Dhama, Khulud Alsalman, Zainab Alalawi, Ziyad Aljofan, Alya Al Mutairi, Mohammed Alomari, Mansour Awad, Awad Al-Omari

**Affiliations:** 1 Research Center, Almoosa Specialist Hospital, Al-Ahsa 31982, Saudi Arabia; abbas4080@hotmail.com (A.A.M.); g.yousif@almoosahospital.com.sa (G.Y.A.); 2 College of Nursing, Princess Norah Bint Abdulrahman University, Riyadh 12214, Saudi Arabia; 3 School of Nursing, University of Wollongong, Wollongong, NSW 2522, Australia; 4 Department of Intensive Care, Almoosa Specialist Hospital, Al-Ahsa 31982, Saudi Arabia; Research.center@almoosahospital.com.sa; 5 Administration of Pharmaceutical Care, Al-Ahsa Health Cluster, Ministry of Health, Al-Ahsa 31982, Saudi Arabia; saalhumaid@moh.gov.sa; 6 Molecular Diagnostic Laboratory, Johns Hopkins Aramco Healthcare, Dhahran 31982, Saudi Arabia; arabaan@gmail.com; 7 Department of Public Health and Nutrition, The University of Haripur, Haripur 22610, Pakistan; 8 Department of General Surgery, Alomran General Hospital, Al-Ahsa 36358, Saudi Arabia; Mohammed.abdulmajeed.gh@hotmail.com; 9 School of Medicine, RCSI Bahrain, Busaiteen 15503, Bahrain; hiba.chagla1@gmail.com; 10 Department of Medicine Keystone Health, Penn University School of Medicine, Hershey, PA 16801, USA; drraghutg@gmail.com; 11 Geisinger Community Medical Center, Geisinger Health System, Scranton, PA 18510, USA; asharma1@geisinger.edu; 12 Division of Pathology, ICAR-Indian Veterinary Research Institute, Izatangar, Bareilly 243122, Uttar Pradesh, India; kdhama@rediffmail.com; 13 AlJaber Hospital for Eye & ENT, Ministry of Health, Al-Ahsa 31973, Saudi Arabia; k.abdullah_99@hotmail.com; 14 Division of Allergy and Immunology, College of Medicine, King Faisal University, Al-Ahsa 31982, Saudi Arabia; zalalwi@kfu.edu.sa; 15 College of Medicine, King Saud University, Riyadh 54321, Saudi Arabia; ziyadaljofan@gmail.com; 16 Department of Mathematics, Faculty of Science, Taibah University, Medina 54321, Saudi Arabia; amutairi@taibahu.edu.sa; 17 Palliative Care Department, King Fahad Medical City, Riyadh 12214, Saudi Arabia; moualomari@gmail.com; 18 Commitment Administration, General Directorate of Health Affairs, Ministry of Health, Medina 42351, Saudi Arabia; 19 Research Center, Dr. Sulaiman Al Habib Medical Group, Alfaisal University, Riyadh 12214, Saudi Arabia; Research.Center@drsulaimanalhabib.com

**Keywords:** COVID-19, severe acute respiratory syndrome coronavirus 2, infectious disease, critical cases, intensive care units, survival, Saudi Arabia

## Abstract

*Backgroundand Objectives*: COVID-19 is a novel infectious disease caused by a single-stranded RNA coronavirus called severe acute respiratory syndrome coronavirus 2 (SARS-CoV-2). We aimed to conduct a nationwide multicenter study to determine the characteristics and the clinical prognostic outcome of critically ill COVID-19 patients admitted to intensive care units (ICUs). *Materials and Methods*: This is a nationwide cohort retrospective study conducted in twenty Saudi hospitals. *Results*: An analysis of 1470 critically ill COVID-19 patients demonstrated that the majority of patients were male with a mean age of 55.9 ± 15.1 years. Most of our patients presented with a shortness of breath (SOB) (81.3%), followed by a fever (73.7%) and a cough (65.1%). Diabetes and hypertension were the most common comorbidities in the study (52.4% and 46.0%, respectively). Multiple complications were observed substantially more among non-survivors. The length and frequency of mechanical ventilation use were significantly greater (83%) in the non-survivors compared with the survivors (31%). The mean Sequential Organ Failure Assessment (SOFA) score was 6 ± 5. The overall mortality rate of the cohort associated with patients that had diabetes, hypertension and ischemic heart disease was 41.8%. *Conclusion*: Age; a pre-existing medical history of hypertension, diabetes and ischemic heart disease; smoking cigarettes; a BMI ≥ 29; a long mechanical ventilation and ICU stay; the need of ventilatory support; a high SOFA score; fungal co-infections and extracorporeal membrane oxygenation (ECMO) use were key clinical characteristics that predicted a high mortality in our population.

## 1. Introduction

Consequences of COVID-19: COVID-19 is associated with many diverse outcomes. Severely ill patients develop acute respiratory distress syndrome (ARDS) and severe cardiovascular and renal complications that may lead to death. A recent study conducted in Saudi Arabia found that the presentation of COVID-19 varied within the population, ranging from mild cases with less abundant symptoms such as olfactory loss, weight loss, diarrhea and headaches [1] to severe cases with patients suffering from chest pain, a shortness of breath (SOB) and even loss of speech or movement [2,3]. Severely ill patients with COVID-19 are more likely to be admitted to the ICU [4]. Previously published reports showed that the percentage of patients with a severe illness who required an ICU admission was reported to be between 4% and 32% and the ICU capacity had become heavily burdened by policy considerations [5].

Characteristics affecting COVID-19 outcomes: The severity and outcome of a COVID-19 infection are dependent on both the virus itself and the host’s immune response [6]. Research from numerous countries such as China, Italy, Sweden and the United States have linked a poor prognosis of the disease with factors such as increased age, male gender and pre-existing chronic diseases such as high blood pressure, cardiovascular disease and diabetes [3]. At the same time, the pediatric cases have been shown to have a milder clinical illness course [6,7,8,9]. Multiple schemes have been proposed to explain the current rate of COVID-19 infection and mortality including stricter measures, better health care systems and genetic factors, among others [10]. A particular hypothesis focused on the genetic variants within the angiotensin-2 converter gene (ACE2) [10]. The ACE2 receptor acts as an entry point for coronavirus and is responsible for causing the dysregulated hyperimmune response in COVID-19. Various studies have suggested that the variability within the ACE2 gene can affect the virus’s entry into the cell of the host and, hence, the extent of organ damage [11]. A previous study showed that one variable within the ACE2 (N720D) gene was responsible for lower infection rates in the Middle East compared with Europe. Previous studies have shown that many countries around the world have been affected differently by the COVID-19 infection, ranging from a high incidence and high mortality rates in countries such as the United States, France and Spain to a low incidence and mortality rates in countries such as New Zealand [8,9]. These findings indicate the importance of investigating different populations around the world to gain a greater insight into the disease and to understand the impact of COVID-19 among different regions [8] as well as enabling us to be scientifically better equipped to handle similar future emergencies [12,13]. The present study aimed to conduct a nationwide multicenter study centered upon severely ill COVID-19 patients admitted to intensive care units (ICUs) in Saudi Arabia, assessing their clinical characteristics and prognosis. The results of this study can be used to identify the risk factors associated with a poor prognosis in COVID-19 patients in Saudi Arabia and help develop preventive measures to decrease the disease burden. In addition, the results from this study can help improve patient resource allocation through the risk stratification of COVID-19 patients.

## 2. Materials and Methods

This was a nationwide multicenter cohort retrospective study conducted in twenty tertiary public and private Saudi hospitals to examine the survival of patients with COVID-19 influenced by multiple demographic and clinical characteristics. Research Electronic Data Capture (REDCap)—a web-based software tool developed by Vanderbilt University that allows researchers to create secure online case report forms for data capture, management and analysis—was used to collect the required data on all targeted COVID-19 patients by each research coordinator at the participating hospitals under the supervision of the primary investigator physician. From each hospital, the following data of ICU patients were collected: the socio-demographic profile characteristics of the patients admitted to the ICU with a COVID-19 infection, the laboratory parameters, therapeutic interventions, invasive and non-invasive mechanical ventilation settings and modes, complications and the outcomes of patients.

The following ICU admission criteria were utilized to recruit the current study sample: an ICU patient with a confirmed SARS-CoV-2 infection and (1) unstable vital signs, (2) unstable hemodynamic monitoring, (3) potentially requiring mechanical ventilation support, (4) respiratory arrest, (5) organ failure, (6) potentially requiring continuous renal replacement support (CRRT), (7) abnormal ECG findings, (8) acidosis, (9) a decreased level of consciousness and (10) potentially requiring vasopressor support. Information sources were the files of patients, electronic health information system records and the laboratory reports of patients. The patients were stratified based on the treatment outcome (survival and non-survival). Statistical Package for Social Sciences (SPSS, version 25) was used for the data analysis. The collected data were validated for accuracy and completeness before the statistical analysis. We performed a detailed descriptive analysis for the socio-demographic and clinical variable reporting frequencies and the means ± SD. At the same time, inferential statistics were conducted, applying chi-squared and multiple logistic regression tests. The differences between the treatment groups were considered statistically significant when the two-sided *p*-values ≤ 0.05. A Kaplan–Meier survival analysis method was employed to compare the survival times between patients with different confounding variables as well as for the logistic regression.

## 3. Results

A total of 1740 intensive care COVID-19 patients were collected and analyzed. A univariate analysis for several of the socio-demographic and clinical characteristics of the critically ill patients with COVID-19 are shown in Table 1 including sex, nationality, age, BMI, coexisting morbidities, a few laboratory findings and ECMO use. The majority (1085; 73.8%) were male; half of them (727; 49.5%) were Saudi nationals with a mean age of 55.9 ± 15.1 years and a mean body mass index (BMI) 30.1 ± 6.8 kg/m^2^. A total of 770 (52.4%) participants had a history of diabetes and 676 (46.0%) had hypertension as a comorbidity. The most common presenting symptoms were a shortness of breath (SOB) in 1196 (81.3%) followed by a fever in 1084 (73.7%) and a cough in 957 (65.1%). Fatigue, a sore throat, muscle ache, a headache and chest pain were the next frequent and the least frequent symptoms were abdominal pain, a loss of taste and a loss of smell. A total of 426 (29.0%) patients required mechanical ventilation through their hospital stay. The mean Sequential Organ Failure Assessment (SOFA) score was 6 ± 5. The overall mortality rate was 614 (41.8%) with a significantly higher rate among patients with DM, HTN and ISH comorbidities (Table 1).

The results showed several statistically significant differences in the demographic and clinical characteristics that influenced the survival rate such as nationality—the rate of death among non-Saudi patients was significantly higher than the rate of death among Saudi patients (351 (57.2%) vs. 263 (42.8%), *p*-value 0.0001)—and the mean Sequential Organ Failure Assessment (SOFA) score, which was significantly higher in non-survival patients ((7 ± 4) vs. (4 ± 3), *p*-value 0.0001). Table 2 presents a group of complications including pulmonary embolism (PE), deep venous thrombosis (DVT), a stroke and a pneumothorax, which were observed significantly more among non-survivors. The results also revealed a significant association between positive respiratory cultures and a poor survival (25% vs. 9% (*p*-value 0.0001)). Interventional invasive mechanical ventilation use was significantly greater among non-survivors (83% vs. 31% of survivors (*p*-value 0.0001)). Table 2 also shows that the duration of mechanical ventilation was significantly shorter (6.3 vs. 12.2 days, *p*-value 0.001) among survivors.

**Figure 1:**
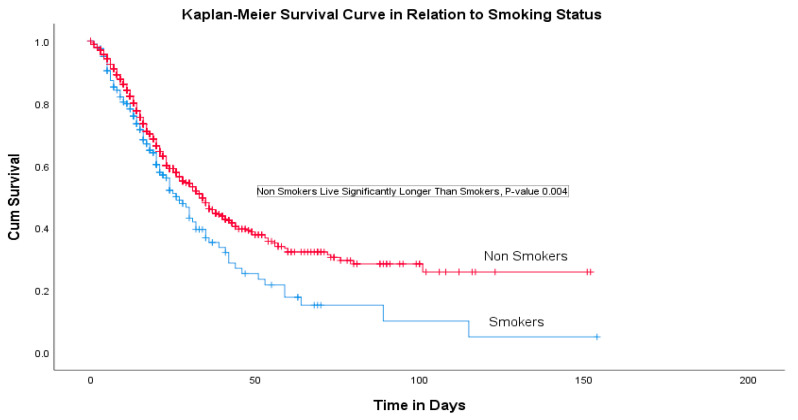
Survival analysis.

A multivariate logistic regression analysis indicated that the patient’s age, nationality, ethnicity, duration of mechanical ventilation, SOFA score and presence of ischemic heart disease (IHD) were significant predictors for the survival rate (Table 3). COVID-19 patients with coexisting IHD had a 70% higher risk of death compared with others (OR 1.72 (95% CI, 1.10–2.69), *p*-value 0.02). There were 196 (13.7%) cigarette smokers and the Kaplan–Meier curve revealed a significantly higher survival among non-smokers with COVID-19 with a *p*-value of 0.004 (Figure 1).

## 4. Discussion

This retrospective cohort study was conducted in twenty Saudi hospitals where 1740 intensive care COVID-19 patients were recruited. The study indicated that the highest rates of presenting symptoms were a shortness of breath (SOB) in 1196 (81.3%) patients followed by a fever in 1084 (73.7%) and a cough in 957 (65.1%). Fatigue, a sore throat, muscle ache, a headache and chest pain were found to be less common and abdominal pain, a loss of smell and a loss of taste were the least common presenting complaints. This finding is similar to previous studies; however, the lower rates of the loss of smell and taste have been previously suggested to be a result of underreporting and neglect by those who suffer from a severe infection [14,15,16,17,18]. Our study used multiple logistic regression and discovered that age, nationality and ethnicity, the duration of mechanical ventilation, SOFA score and the presence of IHD were significant predictors of the survival rate. We found a statistically significant difference in several demographic and clinical characteristics that influenced the survival rate significantly. The current study showed that most cases were male patients (73.8%) and half of them (49.5%) were Saudi nationals with a mean age of 55.9 ± 15.1 years. It was also found that patients with cardiovascular disease, hypertension and COPD were at a high risk of a severe illness and ICU admission. A systematic review and meta-analysis that included seven studies (1813 COVID-19 patients) demonstrated that ICU patients were older, with an average of 62.4 years of age, compared with non-ICU patients (46 years), with a greater proportion of male patients. Additionally, we found that 1591 predominantly older, male patients with comorbid conditions admitted to ICUs had moderate to severe ARDS [16,19]. The overall mortality rate in our study was 614 (41.8%) and the rate of mortality was significantly higher in patients with DM, HTN and ISH comorbidities [14]. The COVID-19 patients with coexisting IHD had a 70% higher risk of death compared with the others. The increased incidence and severity of COVID-19 among older male patients could be explained because of the high rate of comorbidities (such as hypertension) in older males [13,20,21]. Furthermore, the link between hypertension and COVID-19 severity is also linked to the use of ACE inhibitors as ACE2 serves a role in SARS infections [22]. Among our cohort of critically ill COVID-19 patients, 196 were cigarette smokers (13.7%) and the Kaplan–Meier curve revealed a significantly higher survival among non-smoking patients with COVID-19 (*p*-value 0.004). This finding is in accordance with previously published research [18] and emphasizes the need to prevent smoking amongst the population to safeguard against adverse outcomes.

A previously conducted study showed different mortality rates and associated risk factors. For instance, in China, they found that among 226 COVID-19 critical care patients, 87 (38.5%) died [23]. In Spain, of the total 237 critically ill COVID-19 patients, 55 died and 116 remained in the ICU [21]. In a large multicenter prospective study (*n* = 4224) conducted in Belgium, France and Switzerland, the mortality rate was 31% on day 90 of ICU admission [23]. Another study in Spain and Andorra [22] reported a mortality rate of 31%. In Sweden, of the 260 participants, mortality was found in 60 of the ICU patients [24] and there was a 35% mortality rate in ICU patients [23,24,25]. The mortality rate was 18.4–40.4% among severely ill patients with COVID-19 at day 30 after admission in the United States and 22% in Australia [26,27,28,29,30]. According to our results, complications such as a pulmonary embolism and pneumothorax have been linked to higher mortality rates among patients with COVID-19. Our results showed that a PE was diagnosed in 20 (2.3%) survivors and in 24 (3.9%) non-survivors. This finding was consistent with a recent study performed in Germany that conducted full body autopsies on 26 patients who had died after being infected with SARS-CoV-2. According to the study, almost one in every four patients developed a PE likely because of a DVT throughout the course of the disease. However, even though the incidence of a PE was higher amongst non-survivors, it was found that a PE was the immediate cause of death in only two cases. This was hypothesized to be due to the use of anticoagulants. This also suggests the need to further investigate the role anticoagulation has on reducing COVID-19-related deaths [11,31]. Among the COVID-19 patients involved in our study, 508 (82.7%) non-survivors required MV compared with the 270 (31.5%) survivors requiring MV. A total of 19 (2.2%) of the survivors and 70 (11.4%) of the non-survivors suffered from a pneumothorax. Our findings are supported by previous studies where although the incidence of a pneumothorax was low (0.3%) in admitted COVID-19 patients, this incidence increased up to 23.8% in those requiring MV. In addition to this, the time interval between a pneumothorax diagnosis and admission had been observed to be around 9 to 19.6 days whereas the time between MV initiation and a pneumothorax was noted to be 5.4 days [32,33,34,35]. Although a COVID-19-related pneumothorax can be a result of COVID-19-induced inflammation and immune dysregulation, our findings suggest an association between the use of invasive mechanical ventilation and a pneumothorax and encourage further studies to be performed to assess this association. Several limitations should be taken into full consideration when interpreting the current study findings. This is only a retrospective short-term observational study design; a prospective research design would give us greater insight into the outcomes of severe COVID-19 infections admitted to the ICU. It is possible that there was a selection bias although the sample was determined by a multidisciplinary ICU team. Additionally, the follow-up was limited to the November of 2020, hindering the possibility of including all outcomes. Subsequently, there may have been a certain partiality regarding the prognosis of the patients as several data were not in the case review form, which prevented us from conducting a further analysis. The clinical follow-up data for patients after recovery from a COVID-19 infection could be used to examine the long term physical and psychological abnormalities.

## 5. Conclusions

A COVID-19 infection is associated with a variety of different outcomes. According to our study, the most common presenting symptoms among our study population included a shortness of breath, a fever and a cough. The predictors of mortality in our population included patient factors such as such as age, BMI > 29, cigarette smoking and the presence of comorbid conditions such as hypertension, diabetes and ischemic heart disease. Other factors affecting the prognosis also included a long ICU stay, mechanical ventilation, a high SOFA score, fungal co-infections and ECMO. Our study suggests the proper evaluation of the need for mechanical ventilation or ECMO on a case-by-case basis. Increasing the awareness about key factors involved in the prognosis of a COVID-19 infection improves the clinical outcomes by ensuring timely and correct resource allocations as well as allowing preventative measures to be put into place. Future randomized trials are needed to confirm or dispute the reported observations in the current study.

## Figures and Tables

**Table 1:** Baseline characteristics (*n* = 1470).

Characteristics	Survival (856; 58.2%)	Non-Survival (614; 41.8%)	*p*-Value
Gender			
Male	629 (73.5%)	459 (74.8%)	
Female	227 (26.6%)	155 (25.2%)	0.58
Nationality			
Saudi	464 (54.2%)	263 (42.8%)	
Non-Saudi	392 (45.8%)	351 (57.2%)	0.0001
Presenting symptoms			
Shortness of breath	700 (81.8%)	496 (80.8%)	0.85
Fever	644 (75.2%)	440 (71.7%)	0.25
Cough	561 (65.5%)	396 (64.5%)	0.1
Fatigue	174 (20.3%)	105 (17.1%)	0.16
Sore throat	137 (16.0%)	092 (15.0%)	0.73
Muscle ache	109 (12.7%)	071 (11.9%)	0.75
Headache	115 (13.4%)	060 (9.8%)	0.05
Diabetes mellitus comorbidity			
Yes	429 (50.1%)	341 (55.5%)	
No	427 (49.9%)	273 (44.5%)	0.04
Hypertension comorbidity			
Yes	367 (42.9%)	309 (50.3%)	
No	489 (57.1%)	305 (49.7%)	0.005
Ischemic heart disease comorbidity			
Yes	85 (9.9%)	99 (16.1%)	
No	771 (90.1%)	515 (83.9%)	0.0001
Bronchial asthma comorbidity			
Yes	83 (9.7%)	45 (7.3%)	
No	773 (90.3%)	569 (92.7%)	0.11
CKD comorbidity			
Yes	64 (7.5%)	59 (9.6%)	
No	792 (92.5%)	555 (90.4%)	0.15
ICU length of stay			
≤ 28 days	644 (75.2%)	517 (84.2%)	
29–60 days	212 (24.8%)	97 (15.8%)	0.0001
Culture			
Positive candidiasis	12 (1.4%)	25 (4.1%)	
Negative candidiasis	844 (98.6%)	589 (95.9%)	0.001
Inflammatory markers			
Erythrocyte sedimentation rate (ESR)	48.71 ± 48.35	49.21 ± 34.77	0.93
C-reactive protein (CRP)	142.73 ± 193.91	131.30 ± 211.89	0.64
Ferritin	2496.69 ± 2874.89	1428 ± 1774	0.21
Duration of mechanical ventilation	6.91 ± 14.07 days	12.74 ± 13.90	0.0001
SOFA score	4 ± 3	7 ± 4	0.0001
Age	54.05 ± years	58.43 ± 15.42	0.0001
BMI	30.48 ± 7.10	29.60 ± 6.34	0.02

CKD: chronic kidney disease; ICU: intensive care unit; SOFA: Sequential Organ Failure Assessment; BMI: body mass index.

**Table 2:** Intervention and overall outcomes of patients (*n* = 1470).

Intervention	Survival (856; 58.2%)	Non-Survival (614; 41.8%)	*p*-Value
HFNC			
Need HFNC	310 (36.2%)	136 (22.1%)	
Did not need HFNC	546 (63.8%)	378 (77.9%)	0.0001
BIPAP			
Need BIPAP	90 (10.5%)	115 (18.7%)	
Did not need BIPAP	766 (89.5%)	499 (81.3%)	0.0001
Invasive mechanical ventilation			
Yes	270 (31.5%)	508 (82.7%)	
No	586 (68.5%)	106 (17.3%)	0.0001
Duration of mechanical ventilation	6.3 ± 10.7 days	12.2 ± 11.1 days	0.0001
ECMO			
Done	20 (2.3%)	51 (8.3%)	
Not done	836 (97.7%)	563 (91.7%)	0.0001
Paralysis			
Yes	184 (21.5%)	378 (61.6%)	
No	672 (79.5%)	236 (38.4%)	0.0001
Positive respiratory culture			
Yes	76 (8.9%)	151 (24.6%)	
No	780 (91.1%)	463 (75.4%)	0.0001
Complications, PE			
Yes	20 (2.3%)	24 (3.9%)	
No	836 (97.7%)	590 (96.1%)	0.017
Complications, DVT			
Yes	10 (1.2%)	23 (3.7%)	
No	846 (98.8%)	591 (96.3%)	0.0001
Complications, stroke			
Yes	10 (1.2%)	22 (3.6%)	
No	846 (98.8%)	592 (96.4%)	0.001
Complications, pneumothorax			
Yes	19 (2.2%)	70 (11.4%)	
No	837 (97.8%)	544 (88.6%)	0.0001

HFNC: high flow nasal cannula; ECMO: extracorporeal membrane oxygenation; PE: pulmonary embolism; DVT: deep venous thrombosis.

**Table 3:** Multiple logistic regression (*n* = 1470).

Characteristics	OR	95% CI	*p*-Value
Gender			
Male vs. female	1.06	(0.76–1.48)	0.74
Nationality			
Non-Saudi vs. Saudi	2.56	(1.87–3.52)	0.0001
Blood culture for candidiasis			
Positive vs. negative	1.23	(0.55–2.72)	0.63
DM			
Diabetic vs. non-diabetic	1.06	(0.76–1.48)	0.74
HTN			
Hypertensive vs. not hypertensive	1.1	(0.77–1.57)	0.59
IHD			
IHD vs. no IHD	1.72	(1.10–2.69)	0.02
Bronchial asthma (BA)			
BS vs. no BA	0.75	(0.46–1.22)	0.24
CKD			
CKD vs. no CKD	0.8	(0.48–1.34)	0.4
ECMO	1.51	(0.77–2.95)	0.23
SOFA	1.17	(1.12–1.23)	0.0001
Age	1.03	(1.01–1.04)	0.0001
BMI	0.98	(0.96–1.002)	0.08
Duration of mechanical ventilation	1.04	(1.03–1.06)	0.0001

DM: diabetes mellitus; HTN: hypertension; IHD: ischemic heart disease; CKD: chronic kidney disease; ECMO: extracorporeal membrane oxygenation; SOFA: Sequential Organ Failure Assessment; BMI: body mass index.

## Data Availability

Not applicable.
